# A Peer-Led, Social Media-Delivered, Safer Sex Intervention for Chinese College Students: Randomized Controlled Trial

**DOI:** 10.2196/jmir.7403

**Published:** 2017-08-09

**Authors:** Wai Han Sun, Carlos King Ho Wong, William Chi Wai Wong

**Affiliations:** ^1^ Department of Family Medicine and Primary Care The University of Hong Kong Ap Lei Chau, Hong Kong China (Hong Kong)

**Keywords:** sex education, social media, randomized controlled trial

## Abstract

**Background:**

The peer-led, social media-delivered intervention is an emerging method in sexual health promotion. However, no research has yet investigated its effectiveness as compared with other online channels or in an Asian population.

**Objective:**

The objective of this study is to compare a peer-led, social media-delivered, safer sex intervention with a sexual health website. Both conditions target Chinese college students in Hong Kong.

**Methods:**

A randomized controlled trial was conducted with a peer-led, safer sex Facebook group as the intervention and an existing online sexual health website as the control. The intervention materials were developed with peer input and followed the information-motivation-behavioral skills model; the intervention was moderated by peer educators. The participants filled out the online questionnaires before and after the 6-week intervention period. Outcome evaluations included safer sex attitudes, behavioral skills, and behaviors, while process evaluation focused on online experience, online-visiting frequency, and online engagement. The effect of online-visiting frequency and online engagement on outcome variables was investigated.

**Results:**

Of 196 eligible participants—100 in the control group and 96 in the intervention group—who joined the study, 2 (1.0%) control participants joined the Facebook group and 24 of the remaining 194 participants (12.4%) were lost to follow-up. For the process evaluation, participants in the intervention group reported more satisfying online experiences (P<.001) and a higher level of online-visiting frequency (P<.001). They also had more positive comments when compared with the control group. For outcome evaluation, within-group analysis showed significant improvement in condom use attitude (P=.02) and behavioral skills (P<.001) in the intervention group, but not in the control group. No significant between-group difference was found. After adjusting for demographic data, increased online-visiting frequency was associated with better contraceptive use behavioral intention (P=.05), better behavioral skills (P=.02), and more frequent condom use (P=.04).

**Conclusions:**

A peer-led, social media-delivered, safer sex intervention was found to be feasible and effective in improving attitudes toward condom use and behavioral skills, but was not significantly more effective than a website. Future research may focus on the long-term effectiveness and cost-effectiveness of this popular method, as well as the potential cultural differences of using social media between different countries.

**Trial Registration:**

Chinese Clinical Trial Registry (ChiCTR): ChiCTR-IOR-16009495; http://www.chictr.org.cn/showprojen.aspx?proj=16234 (Archived by WebCite at http://www.webcitation.org/6s0Fc2L9T)

## Introduction

### Youth Sexual Health and Education

The sexual and reproductive health of young people constitutes a major health concern, as they are at risk of unwanted pregnancy [1] or of contracting sexually transmitted infections (STIs) [2]. Indeed, unsafe sex and the lack of contraception were the second- and fourth-most common risk factors for cause-specific, disability-adjusted life years for young people globally [3]. In China, the prevalence of condom use among sexually active college students is low at 49.7% [4]; more alarmingly, over 40% of students reported having either impregnated a girlfriend or having an unwanted pregnancy [5]. In Hong Kong, chlamydia prevalence was high in sexually active young women and men, at 5.8% and 4.1%, respectively [6]. A study of Chinese college students also showed poor sexual health knowledge and negative attitudes toward contraception [5]. Similar to their western peers, Chinese young people appear to have less than optimal knowledge, attitudes, and behaviors in relation to safer sex practice. In Hong Kong, no mandatory sex education is required in schools, although it is sometimes provided within extracurricular activities or on an ad hoc basis at the discretion of individual institutions. Instead, most young people learn about sex from their peers, from social workers, or from the Internet [7].

Safer sex promotion via sexual health education has been found to successfully increase sexual health knowledge and change attitudes and behaviors among teens and young adults [8,9]. Among them, a number of trials of the information- motivation-behavioral (IMB) skills model found information on risk reduction, motivation, and behavioral skills to be fundamental determinants in changing HIV risk behaviors [10]. Globally, safer sex interventions targeting college students combined both IMB model and peer-led approaches, with favorable outcomes [11-13]; for instance, the frequency of keeping condoms at hand increased, as did condom use [11,14,15]. Furthermore, peer-led sex education is a popular approach in youth sex education, as it is believed that young people are more easily influenced by their peers [16]. As found in a systematic review and meta-analysis, which examined a whole spectrum of peer-led sex education studies in more-developed countries, peer-led sex education was effective in changing sexual health knowledge and attitudes among young people [17].

### Social Media as a New Tool

The Internet provides a unique opportunity for safer sex promotion due to its accessibility, confidentiality, and potential for creating personalized messages [18]. As the Internet has become more popular, the amount of research relating to online youth sex education has increased. While there was research on the effectiveness of sexual health promotion through short message service (SMS) text messaging, websites, and online games targeting young people [19-22], research into the use of social media on sex education is mainly exploratory in nature and focuses on process evaluation [23,24]. As young people nowadays spend a large amount of their time online on social media [25], and sexual health has been reported as the most frequently searched topic by young people [23,26], further research is needed to evaluate the effectiveness of social media in promoting safer sex among young people. Furthermore, a systematic examination of sexual health promotional activities on the Internet identified 178 activities on social networking sites [27] and revealed that social media provided valuable peer and social support due to its interactive features [23]. In addition, trained peer educators’ involvement in the development and implementation processes adds value to the interventions [28].

Youth sexual health promotion via social media has many advantages, namely, its popularity among young people; its nature of interactivity, large potential reach, and instant reaction; as well as its facilitation of the use of multimedia materials. This can increase participants’ online engagement and provide a new way of learning, which could bring about safer sex behaviors effectively. Combined with the peer-led approach, this form of online sex education further allows tailor-made content for a large target population involving the consumers and the empowering of youth. These characteristics may contribute to a more successful intervention as it reaches more young people and makes the content more relevant to young people. As shown in previous research, this approach could improve offline safer sex behaviors [28,29].

To the authors’ knowledge, none of the previous research has compared the effectiveness of sexual health promotion delivered on social media with its effectiveness when delivered via other online channels. An active control group is needed to investigate the relative effectiveness of a social media-delivered intervention and other existing digital interventions targeting youth, such as websites or SMS text messaging. This research compares a social media-delivered intervention with an existing sexual health website, as websites have been shown to be effective in bringing about safer sex behaviors [30]. Apart from the mode of delivery, the content of the website is expert led rather than peer led. Moreover, previous trials were conducted in developed countries, such as the United States or Australia, and none have been conducted in Asia. It is therefore unclear whether the findings could be generalized across cultures.

### Study Aims

This study aimed to evaluate the effectiveness of a peer-led, safer sex, social media-delivered intervention among Chinese college students in Hong Kong. Social media has extra benefits for health promotion as compared to traditional websites; for example, it allows peer-to-peer discussion [23]. Among the few evaluative studies on safer sex interventions through social media using the peer-led approach, positive results were evident, such as increasing condom use and increasing STI-testing behaviors [28,29]. Moreover, peer-led approaches were found to be more effective than adult-led approaches in previous literature reviews [17,31]. A sexual health website with expert-led material that did not involve the use of social media was used as the control in this study.

The first hypothesis was that the intervention would have a larger positive effect than would the control on attitudes, behavioral skills, and behaviors in relation to safer sex. The second hypothesis was that the intervention group would have a more positive online experience and higher online-visiting frequency than the control group. The third hypothesis was that the higher the online usage and engagement, the larger the effect the intervention would have on the outcome measures.

## Methods

### Design

A randomized controlled trial with two arms was conducted on college students using a peer-led, safer sex intervention; the trial was developed with active youth input based on the IMB model and conducted via social media in the form of a Facebook group. An existing online sexual health website was used as the control condition.

### Participants and Sample Size

The participants were recruited from three different colleges in Hong Kong. The three universities include two large-scale universities and one small-scale university in different districts of Hong Kong. This enabled the recruitment from a large student base with a wide range of disciplines and geographic locations. The research was promoted to the students through mass emails, student societies, posters around campus, and social media. The eligibility criteria included being an undergraduate student and aged under 25 years.

A previous study on safer sex motivation, which used the same validated scale, showed that the effect size was medium (ie, 0.5) [32]. With the confidence level set at 95% and power as 0.9, the sample size calculation yielded a sample size of 172 [33]. As previous studies indicated that the dropout rate in online research could be as high as 15% [34,35], the final number estimated was 99 individuals in each group.

### Questionnaire and Procedures

The participants signed up voluntarily through an open online survey website, SurveyMonkey, and eligibility questions were asked to screen participants. Screened participants were given information about the research online and they gave their informed consent before they filled in the self-administered survey. The information included a brief introduction to the study, a description of the targeted group, and the benefits and risks of joining the study. The participants were reminded that they could always contact the research team through email or phone if they had any questions or if they came to any harm during the study. Multiple identities were prevented by the survey software prohibiting multiple submissions from the same IP address and through email confirmation. Researchers also sent out the postintervention survey through email. Apart from process and outcome evaluations, the survey also collected sociodemographic information, including age, gender, sexual orientation, and the number of romantic relationships. There were 33 items in total on seven screen pages. A completeness check was applied, but participants were not able to review and change their answers. At the end of the survey, the online survey software randomized the participants into one of the two groups. Our online survey software did the random assignment by allowing the researcher to set a certain percentage for each group. In this study, 50% of the participants were randomized into the intervention condition, while the other 50% were randomized into the control condition. The collected data were protected by password and could only be accessed by the researchers through the institute computer. The usability and technical functionality of the online questionnaire was tested before launching. By completing the baseline and postintervention surveys, participants would receive HK $100 (US $1=HK $7.80) as an incentive.

### Intervention

The intervention material was developed by a group of trained peer educators following the three elements in the IMB model: safe sex knowledge, motivation, and behavioral skills. The details of the peer input are described below. The peer-led intervention was delivered through Facebook, the most popular social media platform among Hong Kong college students. The research team from the host university collaborated with a local community-based organization, Sticky Rice Love, to develop the intervention material with their young educators. This nonprofit organization has been promoting sexual health to Hong Kong youth on social media since 2014, following a peer-led approach. They recruited young people as educators and empowered them to create online sex education material. They have thousands of followers on their Facebook and Instagram profiles.

The peer educators were recruited through Sticky Rice Love in October 2015; they went through training followed by a final assessment in April 2016. The selection criteria included a high attendance in the training sessions, ample time available to be involved in the program, passion toward sex education, accurate sexual health knowledge, and being nonjudgmental when conducting sex education. There were six training sessions covering topics relating to (1) attitude of a sex education provider, (2) sexual violence, (3) female sexual health, (4) male sexual health, (5) safe sex and STIs, and (6) communication skills in a relationship. They were conducted by the Sticky Rice Love staff, a doctor, and a social worker in the specific field. Training activities included direct teaching, interactive games, role play, question sessions, videos, and condom demonstration. There were 19 youth educators with a mean age of 22.5 years (range 19-24) and slightly more female members (11/19, 58%).

The peer educators attended a session about the IMB model in June 2016. They then created the intervention material at their monthly meetings. They had three meetings to finalize the intervention material. The educators were responsible for the selection of the intervention content, presentation, and production. The content included pregnancy, STIs, and safe sex practices (eg, abstinence, condom use, and pills) in the form of text, images, GIFs, and videos. The participants were able to “like” or comment on the Facebook posts. The researchers (WCWW and WHS) examined the information and reminded the peer educators of any missing aspect from the IMB model. One of the investigators (WCWW), who is a physician with over 15 years of sexual health research experience, provided professional input and advice on its development and ensured that the sexual health information was correct and accurate.

The intervention lasted for 6 weeks, from October to November 2016, and the frequency of posts was three to four per week, totaling 21 posts. The intervention was delivered to a “secret” Facebook group created purely for this project. A Facebook group refers to a page in Facebook where group members can post on the wall and interact through discussion threads. As the privacy setting was set to “secret,” only group members could see the group and know who else was in the group. Only people who were invited could join the group. This was used as it could provide a closed network for research purposes. In addition, a Facebook group lets the researchers record the online usage data of each participant, which could be used as an objective process measure. The participants would receive Facebook notifications for each new post. Peer educators were added to the group to moderate by posting questions and responding to comments.

### Control

The participants randomized to the control group would be given the sex education website link of the Hong Kong Family Planning Association [36], the most established, government-funded sex education organization in Hong Kong. The website was set up in 2004 and targets young people in its promotion of sexual health online. A range of information related to safe sex, including contraception and STIs, is included on the website with credible sources, which is similar to the intervention in terms of content. The website is the most well-regarded existing mode of delivery for safe sex information currently available to college students. It serves as an online promotion of sexual health without the peer-led and social media elements and belongs to the Web 1.0 stage of the World Wide Web. Weekly emails were sent to the participants to remind them to visit the website.

### Evaluation

#### Process Measures

The online experience of the participants was assessed according to the important elements of online sexual health promotion found in previous qualitative research [30,37]. These elements are (1) credibility, (2) personal relevance, (3) respect for autonomy, (4) comfort to learn, (5) engaging experience, (6) ease of use, and (7) privacy. Seven items were included on a scale from 1 (Strongly Disagree) to 5 (Strongly Agree). An open-ended question was also included to allow the participants to express their feelings toward the safer sex intervention in their own words.

All participants were asked to self-report the frequency with which they visited their assigned online education platform on a 5-point scale, from 1 (Rarely) to 5 (Always). For the intervention group, online engagement was also recorded when a participant reacted or commented on the Facebook group content.

#### Outcome Measures

Following the IMB model, the measured outcomes included motivation, behavioral skills, and behavior. Although information is one of the constructs in the model, it was found to be an inconsistent construct in relation to behavior [38]. It was included at the development stage but not at the evaluation stage. Motivation change included condom use, attitude, and contraceptive use behavioral intention [10]. Outcome measures were assessed through scales adopted from an IMB model-based HIV prevention intervention and were on a scale that ranged from 1 (Strongly Disagree) to 4 (Strongly Agree) [32]. The condom use attitudes scale score included four items that assessed participants’ beliefs about condom use with a reported internal consistency, alpha, at .82. The contraceptive use behavioral intention scale had three items with a reported internal consistency, alpha, at .61.

To measure changes in behavioral skills, we applied the perceived difficulty and ease of condom use scale, which was first validated in 1998 [39] and was used in other IMB model-based studies [15,40]. It is a 5-point scale ranging from 1 (Very Difficult) to 7 (Very Easy), with seven items. The reported internal reliability, alpha, was .91 among college students [15]. For behavioral changes, the frequency of condom use was measured on a 5-point scale ranging from 1 (Never) to 5 (Always). The measures are included in Multimedia Appendix 1.

### Statistical Methods

Data were analyzed using SPSS version 23 (IBM Corp). Differences in demographic characteristics at baseline between the intervention and control group were assessed using chi-square tests for categorical variables—age group, gender, sexual orientation, and sexually active status. Differences in outcome variables at baseline were assessed using independent *t* tests.

For process evaluation, the aim was to examine if there were differences in online experience and frequency of online visiting between intervention and control groups. Quantitatively, independent *t* tests were used to examine the differences between online experience scores and frequency of online visiting. Qualitatively, the opinions of the participants toward the Facebook group and website were summarized to understand any differences between the two conditions.

For outcome evaluation, the aim was to examine whether the kind of promotion had a significant effect on outcome variables between intervention and control groups. Paired *t* tests were used to examine the within-group changes in outcomes. Repeated measures analysis of variance (ANOVA) was used to compare the effectiveness between the two groups using the Time X Treatment analysis. In addition, linear regressions were performed to assess the effect of frequency of online visiting and online engagement on outcome variables, with adjustment for age, gender, and sexual orientation. Participants in the intervention group were divided into low, medium, and high online engagement according to the number of their Facebook reactions, comments, and posts, but their number of views was not counted. Participants in the control group were regarded as a control for online engagement.

All significance tests were two-tailed and the level of significance was set at a *P* value of less than .05. For missing data, the missing value was imputed by an average score of the group at the respective time point. We applied the intention-to-treat principle, that is, we assumed that participants would remain in their initially allocated group until the end of trial.

### Ethical Approval

This research was approved by the Institutional Review Board of the University of Hong Kong, Hospital Authority Hong Kong West Cluster, in August 2016 (No. UW 16-419).

## Results

### Overview

**Figure 1:**
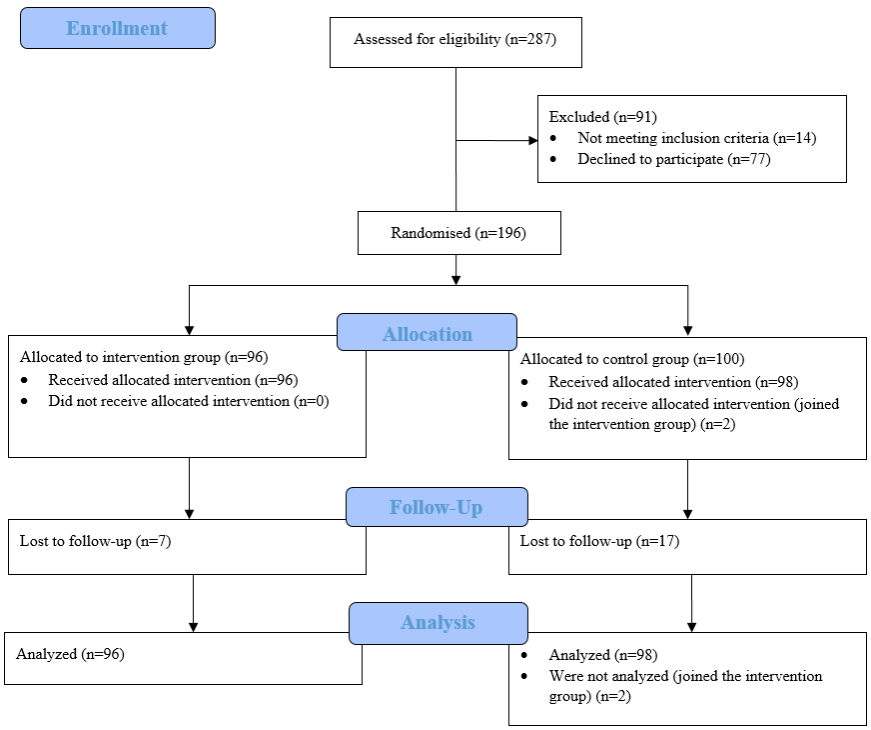
Participant flow diagram.

A total of 287 people initially signed up, while 196 eligible participants completed the preintervention survey and successfully joined the study (see Figure 1) in October 2016; the recruitment period was in September 2016. Participants were randomized to the intervention group (n=96) and the control group (n=100). There were 2 participants in the control group that decided to join the Facebook group; they were subsequently removed from the study. Follow-up was done immediately after the 6-week intervention; 24 participants were lost to follow-up, giving a dropout rate of 12.4% (24/194).

### Baseline Characteristics

Demographic information, including age, gender, sexual orientation, and sexual experience, was requested in the preintervention survey (see Table 1). Participants were mostly aged 19-20 years (85/194, 43.8%), female (131/194, 67.5%), heterosexual (156/194, 80.4%), and sexually inactive (144/194, 74.2%), and most participants never had sex (123/194, 63.4%). The baseline level of the participants’ outcome performances is presented in Table 2. No significant difference between the intervention and control groups at baseline was found.

**Table 1:** Baseline characteristics and outcomes of participants.

Characteristics		Social media intervention group (n=96)	Website control group (n=98)	Total (N=194)	*P*^a^
**Age (years), n (%)**					.67
	17-18	23 (24)	24 (24)	47 (24.2)	
	19-20	41 (59)	44 (45)	85 (44.3)	
	21-22	30 (31)	24 (24)	54 (27.8)	
	23-24	2 (2)	6 (6)	8 (4.1)	
**Gender, n (%)**					.22
	Male	27 (28)	36 (37)	63 (32.5)	
	Female	69 (72)	62 (63)	131 (67.5)	
**Sexual orientation, n (%)**					.47
	Heterosexual	79 (82)	77 (78)	156 (80.4)	
	Homosexual	9 (9)	7 (7)	16 (8.2)	
	Bisexual	5 (5)	11 (11)	16 (8.2)	
	Uncertain	3 (3)	3 (3)	6 (3.1)	
**Ever had sex, n (%)**					.97
	Yes	35 (37)	36 (37)	71 (36.6)	
	No	61 (64)	62 (63)	123 (63.4)	
	Sexually active	26 (27)	24 (24)	50 (25.8)	.77
**Safe sex attitudes and behavior, mean (SD)**					
	Condom use attitude (range 1-4)	3.15 (0.43)	3.24 (0.47)	3.19 (0.46)	.14
	Contraceptive use intention (range 1-4)	3.04 (0.52)	3.09 (0.68)	3.06 (0.61)	.62
	The perceived difficulty and ease of condom use (range 1-5)	3.26 (0.58)	3.32 (0.57)	3.29 (0.58)	.48
	Condom use frequency (n=50)^b^, (range 1-5)	3.80 (1.38)	3.28 (1.54)	3.54 (1.46)	.22

^a^Chi-square tests or independent *t* tests between intervention and control groups.

^b^For sexually active participants only.

**Table 2:** Process evaluation.

Assessment measure	Social media intervention group, mean (SD)	Website control group, mean (SD)	*P*^a^	Mean difference	95% CI
Online experience score	3.86 (0.49)	3.58 (0.51)	<.001	0.27	0.13-0.41
Online-visiting frequency	3.53 (0.81)	2.47 (0.90)	<.001	1.06	0.82-1.30

^a^Independent *t* tests between intervention and control groups.

### Process Evaluation

As shown in Table 2, the evidence supports the hypothesis that the intervention group would have a significantly better online experience (*P*<.001) and a significantly higher online-visiting frequency (*P*<.001) compared to the control group. The second hypothesis, that the intervention group would have a more positive online experience and more online usage than the control group, was also supported.

Qualitatively, comments were received on both intervention and control conditions. For the intervention group, 42% (5/12) of the comments were positive, 16% (2/12) were negative, and 42% (5/12) were neutral. Participants mentioned that the videos were funny, that some practical knowledge was provided, and that the promotion could be extended as a concurrent program to reach other age groups, such as primary school or secondary school students. Participants also pointed out that the information should be presented in bullet point form to improve readability. One participant expressed the view that the content was “inadequate and too superficial.” A total of 2 participants suggested including more information applicable to sexual minorities. A participant said that, since none of his friends were in the group, he felt safe to interact in the group; otherwise, it would be embarrassing. For the control group, 12% (1/8) of the comments were positive, 76% (6/8) were negative, and 12% (1/8) were neutral. A participant expressed the view that it was easy to get information. A total of 4 participants commented on the layout of the sexual health website and said it was “dull, cluttered, and outdated.” A more user-friendly design and a mobile version were both recommended. Another participant felt that the website targeted older audiences.

### Outcome Evaluation

Paired *t* tests of the intervention group showed that the intervention group had a significant change in condom use attitude (*P*=.02) and behavioral skills (*P*<.001), while there was no significant change in the control group (see Table 3). There was no significant change in contraceptive use behavioral intention and contraceptive use frequency in either the intervention or the control group. The intervention group performed better in changing half of the outcome variables, while the control group did not change any outcome variables significantly. Time X Treatment analysis showed that there were insignificant differences between the two groups, although the intervention group showed a larger improvement. All of the *P* values in the repeated measures ANOVA were larger than .05. The first hypothesis, that the intervention would have a larger positive effect on outcome variables than the control, was thus not supported.

In total, 159 Facebook interactions were recorded among 96 users. During the 6-week intervention period, there were 144 “likes,” 13 comments, and 2 participant-initiated posts. A total of 70% (67/96) of the users were categorized as having low online engagement, 20% (19/96) as having medium online engagement, and 10% (10/96) as having high online engagement. The posts on the Facebook group were viewed, on average, by 77% (74/96) of participants. In addition, the peer educators interacted with the participants through commenting on their blogs. They answered the questions asked by the participants and directed them to further resources or, at times, “liked” some of the posts to show encouragement. After adjusting for age, gender, and sexual orientation, regression analysis showed a significant effect of frequency of online visiting on contraceptive use behavioral intention, behavioral skills, and condom use frequency, but not on condom use attitude (see Table 4). The effect of online engagement on outcome variables was not significant.

**Table 3:** Outcome evaluation.

Outcomes	Social media intervention group			Website control group			Time X Treatment analysis
	Baseline, mean (SD)	Postintervention, mean (SD)	*P*^a^	Baseline, mean (SD)	Postintervention, mean (SD)	*P*^a^	*P*^b^
Condom use attitude	3.15 (0.43)	3.26 (0.44)	.02	3.24 (0.47)	3.31 (0.39)	.15	.54
Contraceptive use intention	3.04 (0.52)	3.15 (0.51)	.08	3.09 (0.68)	3.05 (0.52)	.53	.10
The perceived difficulty and ease of condom use	3.26 (0.58)	3.49 (0.52)	<.001	3.32 (0.57)	3.43 (0.57)	.07	.17
Condom use frequency (n=40)	3.90 (1.34)	4.10 (0.94)	.51	3.47 (1.58)	3.31 (1.49)	.27	.29

^a^Paired *t* test between baseline and postintervention data.

^b^Repeated measures analysis of variance (ANOVA) between the baseline and postintervention data of intervention and control groups.

**Table 4:** Regression analysis on the effects of grouping, online-visiting frequency, and online engagement on outcome variables with adjustment for age, gender, and sexual orientation.

Outcome variables			Beta (regression coefficient)	95% CI	*P*
**Condom use attitude**					
	Online-visiting frequency		.02	-0.08 to 0.13	.66
	**Online engagement (control as reference)**				
		Low	.03	-0.13 to 0.19	.73
		Medium	.08	-0.17 to 0.32	.55
		High	.01	-0.31 to 0.33	.96
**Contraceptive use behavioral intention**					
	Online-visiting frequency		.14	0 to 0.28	.05
	**Online engagement (control as reference)**				
		Low	.01	-0.20 to 0.22	.92
		Medium	.21	-0.11 to 0.54	.19
		High	-.05	-0.46 to 0.37	.83
**The perceived difficulty and ease of condom use**					
	Online-visiting frequency		.16	0.03 to 0.30	.02
	**Online engagement (control as reference)**				
		Low	.01	-0.20 to 0.21	.96
		Medium	-.06	-0.37 to 0.26	.73
		High	-.09	-0.50 to 0.32	.66
**Condom use frequency (n=40)**					
	Online-visiting frequency		.65	0.18 to 1.27	.04
	**Online engagement (control as reference)**				
		Low	-.15	-1.08 to 0.78	.74
		Medium	-.38	-1.58 to 0.82	.52
		High	-.59	-2.32 to 1.14	.50

## Discussion

### Principal Findings

This study evaluated a peer-led, social media-delivered, safer sex intervention against a control group, an existing sexual health website, over a 6-week period delivered to a group of Chinese college students. For the process evaluation, participants in the intervention group reported more satisfying online experiences and a higher online-visiting frequency when compared with the control group. Open-ended answers yielded more positive comments from the intervention group than from the control group. For outcome evaluation, the within-group analysis showed significant improvement in condom use attitude and behavioral skills in the intervention group, but not in the control group. Although the intervention group showed more improvement in outcome variables, no significant between-group difference was found. After adjusting for demographic data, only online-visiting frequency was found to have a significant effect on the three outcome variables: contraceptive use behavioral intention, behavioral skills, and condom use frequency.

### Comparison With Previous Research

Previous peer-led sexual health interventions on social media have reported improved results, including increased condom use [28] and increased STI-testing behaviors [29] compared with control groups. However, each of those two interventions was compared with another Facebook group that was unrelated to sexual health: one focused on news content [28] and the other on general health information [29]. This study compared the intervention with an active control, which was a sexual health website. Furthermore, both of the previous studies targeted high-risk groups, such as men having sex with men [29] and ethnic minorities [28], while this research recruited general college students as participants. Moreover, both of the intervention periods in the previous studies were longer—2 months [28] and 12 weeks [29], respectively—than that of this study. On the other hand, a 1-month safer sex intervention compared with no intervention found a significant increase in knowledge and insignificant results on behaviors [41]. While our study had a longer duration, no statistically significant between-group results as such were found. This may be related to the fact that the other studies had longer-term assessment periods, from 1 to 3 months, while ours only included an immediate postintervention assessment, which renders it unable to capture the attitude or behavioral change over a longer period [29,42].

The three main differences between this study and the previous ones are the comparison groups, postintervention measurement, and target populations. Indeed, previous research showed that computerized Web-based interventions are effective in bringing about safer sex behaviors [15,43,44]. A social media-delivered intervention was not found to be significantly more effective than a Web-based intervention in this study, given that the participants were general college students. Having an active control group with immediate follow-up and targeting a low-risk, general college student group might have contributed to the insignificant between-group results from this study.

Previous studies in the United States and Europe reported that the level of participation [41] and online social network ties [29] enhance the intervention effect. This was not supported by this study, as the results failed to show any effect related to the level of online engagement. The differences in the behavior in the virtual world could be due to the cultural difference between an individualistic culture and a collectivistic culture, as found in previous literature [45]. A study investigating the motivation and patterns in using social media among college students in the United States and South Korea found that there were cultural differences in terms of developing and managing social relationships [46]. US students’ online social networks were nearly five times larger than those of their Korean peers, with a similar amount of time spent on social media. This might imply that the ways they view online relationships are different: US students may tend to use social media primarily for casual relationships, whereas Korean students may require a deeper involvement and commitment to obtain social support [46]. In our study, participants expressed the position that they would not join the study if it was not via a “secret” Facebook group. It showed that they tend to care about how friends view them on social media and engaging in sexual health discussion on social media may hurt their social relationships. Discussion of sexual health issues is a sensitive issue, especially in many Asian communities. Participants expressed concerns about letting others know that they were learning about sexual health online. They may be resistant to openly discussing sexual health online and to forming relevant social ties that may threaten their reputation and impose risks to their existing online relationships. Public social ties may be harder to build in Asian communities than previous research has assumed.

### Social Media and Peer-Led Approaches Performed Better on Process Evaluation

While there were many factors affecting the outcomes, online-visiting frequency was found to be significantly related to the outcomes in both groups. It is important to understand what factors contribute to a higher motivation and interest in visiting the site. Comparing the intervention group to the control group, participants in the intervention group had a significantly higher online-visiting frequency and more satisfying online experience. For online-visiting frequency, the mean difference between the two groups was larger than 1, measured on a 5-point scale. Young people have constant access to their mobile phones nowadays. Fewer and fewer of them visit websites, but more are using them for social media [47]. Therefore, the use of platforms also affects online-visiting frequency. Promoting sexual health on social media facilitates a higher online-visiting frequency, which should lead to a larger intervention effect.

The online experience includes elements that were found to be important to online sexual health promotion, including credibility, personal relevance, respect for autonomy, comfort to learn, engaging experience, ease of use, and privacy. Peer-led approaches and social media delivery are better at addressing young people’s needs and expectations relating to sexual health promotion, resulting in a more positive online experience. Furthermore, the content and presentation of the intervention are more up to date; participants reported that the sexual health website was dull and outdated. The intervention materials were developed with a high level of youth input and the result supports the notion that it better suits young people’s needs than expert-led materials.

The importance of anonymity was mentioned by the participants, which was found to be a common concern in some exploratory research on the feasibility of sexual health promotion on social media [24,37,48]. Young people generally care about their image among peers. Although social media has different privacy settings to let users hide certain types of information, young people still have concerns about discussing sexual health on social media. It is also noted that combining peer-led approaches with social media delivery might counterbalance some of the advantages of social media delivery. For instance, training and maintaining the peer educators could increase the cost of the intervention. The sex education material created may not be applicable to other groups in the population, thus decreasing the reach.

### Practical Suggestions

Firstly, from our experience, the engagement and training of the peer educators is of paramount importance. The promotional materials should be appealing to youth and have the ability to reach out to young people through various channels. Training content should be comprehensive, not only including sexual health knowledge, but also online moderating skills and ethical issues such as cyberbullying. Secondly, continuous supervision and safeguarding of the peer educators cannot be ignored. The nature of online programs is such that peer educators often work alone, so team building is highly recommended to promote bonding and prevent volunteers from dropping out. Regular face-to-face meetings or continuous engagement of peer educators would allow them to share their emotions and any issues encountered during the intervention. Some incentives, whether financial or simply a certificate, can help motivate the peer educators to get more involved and acquire new skills. In our program, the peer educators moderated the online Facebook group by providing positive and constructive feedback with frequent auditing and advice from the investigative team. Last but not least, a balance between exposure and privacy for the participants is stressed for a project delivered on social media. As for Facebook, there are different kinds of privacy settings for groups, pages, and profiles. The peer-led approach has been found to be effective in improving safe sex knowledge and attitudes [17], while social media sexual health promotion leads to behavioral change [28,29]. This study shows that peer-led, social media sexual health promotion improves condom use attitude and condom use behavioral skills, and performs better on process evaluation than a sexual health website. Therefore, this approach is recommended, especially when there is no sexual health-related website available for young people.

### Limitations

This research is subject to several limitations. Firstly, outcome variables rely on self-reported measures. Although validated scales were adopted, there is still potential for bias. Social desirability bias is possible, as the baseline of the outcome variables was high, and some participants even achieved full marks at baseline assessment. Secondly, the intervention time may not be long enough to promote a high level of change. However, a longer intervention duration may lead to a high dropout rate, and previous research did show significant results in a 1-month period [41]. Thirdly, due to constraints on resources, this study lacks follow-up assessments. Behavioral and attitudinal change may need a longer time to be realized. Previous online research targeting young people showed that the dropout rate in a 3-month follow-up measurement was nearly 90% [49]. Due to the limitations on resources, we decided at the time to conduct only the immediate follow-up to assess change from this intervention, acknowledging that some changes might occur sometime after the intervention. Lastly, there was a contamination issue between the control and intervention groups, with some of the students signing up together but then being randomized into different groups. Although the participants were asked to maintain confidentiality on the assignment of groups and the intervention content, 2 of the participants assigned to the control group joined the Facebook group and were removed from the study.

### Conclusions

This randomized controlled trial provides important evidence on an emerging online sexual health approach. A peer-led, social media-delivered, safer sex intervention has been found to be effective in improving condom use attitude and behavioral skills in this study. However, when compared with a website, the improvement was not significant. Future research is recommended to further evaluate the long-term effectiveness and cost-effectiveness of a peer-led, social media-delivered approach against current online sexual health promotion for a more systematic comparison. Online-visiting frequency has been found to be significantly related to most of the outcomes: higher online-visiting frequency lead to better improvement on contraceptive use behavioral intention, behavioral skills, and condom use frequency. The intervention group was found to have a higher online-visiting frequency and better online experience. Sexual health promotion on social media is rapidly developing. More programmers and researchers are interested in this delivery method to reach youth. It is recommended that future research investigate which components of social media are important and that researchers be aware of the possible cultural differences between populations using social networking technology.

## Appendix

Multimedia Appendix 1 Measures used in this research.

Multimedia Appendix 2 CONSORT-EHEALTH checklist V1.6.1 [50].

## Abbreviations

ANOVA: analysis of variance
IMB: information-motivation-behavioral
SMS: short message service
STI: sexually transmitted infection
