# Supplementary material for: A Peer-Led, Social Media-Delivered, Safer Sex Intervention for Chinese College Students: Randomized Controlled Trial
Source: J Med Internet Res. 2017 Aug 9;19(8):e284. doi: 10.2196/jmir.7403 (PMC5569248; doi:10.2196/jmir.7403)
Supplement: Multimedia Appendix 1 [file jmir_v19i8e284_app1.pdf]

Multimedia Appendix 1

Measures Used in this Research

Outcome Measures

**Condom Use Attitude**	Strongly Disagree	Disagree	Agree	Strongly Agree
Sex is not as good with a condom				
I do not have a need to use condoms				
Using condoms means you do not trust your partner				
My partner would react badly if I suggested the use of a condom				
**Contraceptive Use Behavioural Intention**				
If I want to have sex, I will first talk with my partner about using contraception				
I will say 'no' to sex if my partner won't use any contraception				
I will use protection such as condom or contraception pills the next time I have sex				

Scales were adopted from an IMB model-based HIV prevention intervention [29].

**Behavioural Skills**	Very Difficult	Difficul t	Neutra l	Easy	Very Easy
How hard would it be for you to buy condoms?					
How hard would it be for you to be supportive if your sexual partner brought up the topic of condoms to reduce the risk of getting a sexually transmitted infection or pregnancy?					
How hard would it be for you to make safe sex with a latex condom sexually exciting for your partner?					
How hard would it be for you to discuss safe sex (for example, always using latex condoms) with your partner in a nonsexual setting?					
How hard would it be for you to use condoms consistently with your partner every time you have a one-night stand?					
How hard would it be for you to use a condom with your partner while under the influence of alcohol or drugs?					
How hard would it be for you to avoid using alcohol or drugs if you think you might be having sex later?					

The scale is adopted from the perceived difficulty and ease of condom use scale which was used and was first validated in 1998 [36], later was used in other IMB theory-based studies [14, 37].

Sexual Behaviour

Did you ever have sex? YES or NO

If yes, did you have sex last month? YES or NO

If yes, please answer the following questions according to your experience last month.

	Never	Rarely	Sometimes	Often	Always
How frequently do you use a condom?					

Process Measures

**Online experience**	Strongly Agree	Agree	Natural	Disagree	Strongly Disagree
The online content was credible.					
The online content was related to my personal experience.					
The online content respected my autonomy of choosing my sexual health.					
I felt comfortable to learn about sex on the online education platform.					
It was an engaging experience for me to learn about sex on the online education platform.					
The online education platform was easy to use.					
I felt that my privacy was protected during my online learning.					

**Visiting Frequency**	Never	Rarely	Sometimes	Often	Always
How often did you visit the online sex education platform in this 6-week period?					
